# Antibiotic Stewardship Initiatives as Part of the UK 5-Year Antimicrobial Resistance Strategy

**DOI:** 10.3390/antibiotics4040467

**Published:** 2015-10-30

**Authors:** Alan P. Johnson, Diane Ashiru-Oredope, Elizabeth Beech

**Affiliations:** 1 Department of Healthcare-Associated Infection & Antimicrobial Resistance, Centre for Infectious Disease Surveillance and Control, National Infection Service, Public Health England, London NW9 5EQ, UK; 2 Antimicrobial Resistance Programme, Public Health England, London NW9 5EQ, UK; E-Mail: diane.ashiru-oredope@phe.gov.uk; 3 NHS Bath and North East Somerset Clinical Commissioning Group, Bath BA2 5RP, UK; E-Mail: elizabeth.beech@nhs.net; 4 NHS England, Patient Safety Domain 5, 6C Skipton House, London SE1 6LH, UK

**Keywords:** antibiotic stewardship, UK antibiotic resistance strategy, antibiotic guardian

## Abstract

Antibiotic use is a major driver for the emergence and spread of antibiotic resistance. Antimicrobial stewardship programmes aim to improve antibiotic prescribing with the objectives of optimizing clinical outcomes while at the same time minimizing unintended consequences such as adverse effects and the selection of antibiotic resistance. In 2013, a five-year national strategy for tackling antimicrobial resistance was published in the UK. The overarching goal of the strategy is to slow the development and spread of resistance and to this end it has three strategic aims, namely to improve knowledge and understanding of resistance, to conserve and steward the effectiveness of existing treatments and to stimulate the development of new antibiotics, diagnostics and novel therapies. This article reviews the antimicrobial stewardship activities included in the strategy and describes their implementation and evaluation.

## 1. Introduction

Antibiotic resistance poses a global threat to public health and clinical medicine. In the UK, surveillance of antibiotic resistance over several decades using a variety of data sources has provided insight into the prevalence of resistance in a range of pathogens. While in some instances resistance has declined in recent years (e.g., methicillin resistance in *Staphylococcus aureus* (*S. aureus*) or erythromycin resistance in *Streptococcus pneumoniae* from children), in other pathogens, particularly Gram-negative bacteria, resistance to key antibiotics is either rising or remains at unacceptably high levels [1]. In response to the threat posed by resistance, the English government mounted a number of initiatives, including (i) making it mandatory for hospitals to report all cases of bacteraemia caused by *S. aureus* (both methicillin-resistant and methicillin-susceptible) and *Escherichia coli*, and all cases of *Clostridium difficile* (*C. difficile*) infection [2,3,4]; (ii) establishing an expert Advisory Committee on Antimicrobial Resistance and Healthcare-Associated Infections to advise on actions needed to tackle the problem of resistance [5]; (iii) establishing the English Surveillance Programme for Antimicrobial Usage and Resistance (ESPAUR) [6]; (iv) updating relevant legislation, in particular the Health and Social Care Act 2008 (Code of Practice on the Prevention and Control of Infections and Related Guidance) to include a new section on the role of infection prevention in optimizing antimicrobial use and reducing antimicrobial resistance [7]; and (v) publication in 2013 of a five-year national antimicrobial resistance strategy, comprising seven areas for action (Table 1) aimed at controlling and ideally reducing the burden of resistance [8].

**Table 1:** The seven key areas for action in the UK five-year antimicrobial resistance strategy [8].

Key Actions
1. Improving infection prevention and control practices
2. Optimizing prescribing through stewardship
3. Improving professional education, training and public engagement
4. Developing new drugs, treatments and diagnostics
5. Better access to and use of surveillance data
6. Better identification and prioritization of AMR research needs
7. Strengthened international collaboration

The UK five-year national strategy for tackling antimicrobial resistance includes optimising antibiotic prescribing through stewardship (Table 1), although it is worth noting that two of the other action points, namely improving infection prevention and control practices and improving professional education, training and public engagement, strongly overlap with, or can be considered part of the bundle of interventions that might make up an antibiotic stewardship programme. This article reviews a number of stewardship activities implemented in England over the last two years in support of the five-year national strategy for controlling antibiotic resistance.

## 2. Antibiotic Use as a Driver of Antibiotic Resistance

Although the epidemiology of antibiotic resistance is complex, it is recognized that use of antibiotics is a major driver for the emergence and spread of resistant bacteria. This has led to the development and implementation of antimicrobial stewardship programmes, which comprise a range of interventions to improve antibiotic use in order to optimize clinical outcomes, while at the same time minimizing unintended consequences including toxicity, the selection of certain pathogenic organisms (e.g., *C. difficile*) and the emergence and spread of resistance. Stewardship programmes should typically consider a range of issues including monitoring and evaluating antimicrobial prescribing and how this relates to local antimicrobial resistance patterns, together with the provision of regular feedback to prescribers. The National Institute for Health and Care Excellence, as the body responsible for providing evidence-based national guidance and advice to improve health and social care in England, recently issued guidance recommending the establishment of antimicrobial stewardship programmes [9]. Among the key actions highlighted was for all healthcare organizations to establish multi-disciplinary antimicrobial stewardship teams that would have an antimicrobial pharmacist and a medical microbiologist as core members capable of providing expert advice alongside co-opted members who expertise and training reflected the clinical care setting.

### 2.1. Antibiotic Prescribing in Primary Care

**Figure 1:**
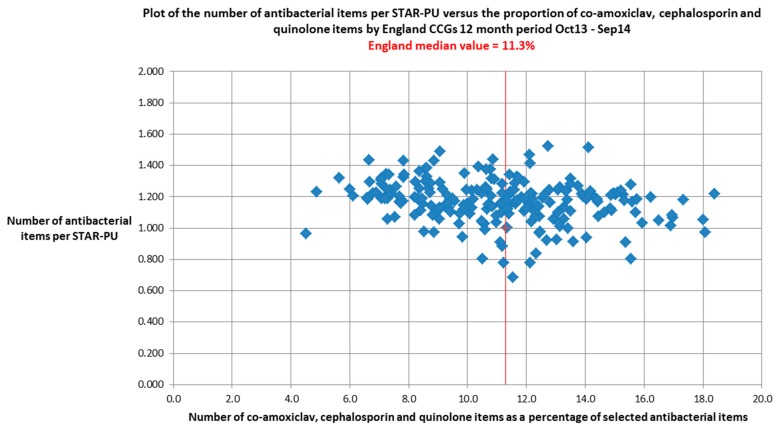
Scatterplot of antibiotic prescribing comparator values (MOKTT) for all 211 Clinical Commissioning Groups in England showing variation in antibiotic prescribing activity [16].

Assessment of the efficacy of antimicrobial stewardship programmes requires the collection and analysis of data on trends in antibiotic prescribing in different settings to determine the impact that implementation of stewardship activities has on the quality and quantity of antibiotic prescribing. England has a state-funded National Health Service (NHS) that provides healthcare free at the point of access, although medicines prescribed in primary care and hospital outpatient settings do have a fixed patient payment. Provision of medical care is overseen by NHS England and is organised through over 200 Clinical Commissioning Groups comprising groups of General Practices that work together to plan and design local health services [10]. Primary care prescriptions are dispensed by community pharmacies and some rural general practitioners with dispensing contracts, with data on medicines use being captured, analysed and published by the NHS Business Services Authority [11]. This process allows the accurate surveillance of antibiotic use in primary care with usage data being available at national, Clinical Commissioning Group and general practitioner level. While the data are available as individual prescription level information for Clinical Commissioning Groups, it is routinely reported as Medicines Optimisation Key Therapeutic Topics (MOKTT) comparators on the NHS Business Services Authority Information Services Portal [12]. These comparators include the number of antibacterial items per STAR-PU (Specific Therapeutic group Age-sex Related Prescribing Units), an age and sex weighted prescribing unit based on general practitioner registered population, and the proportion of broad-spectrum antibiotics (cephalosporins, quinolones and co-amoxiclav) as a percentage of all antibiotics. Use of these comparators allows the identification of variation in use of antibiotics between Clinical Commissioning Groups (Figure 1) and individual general practitioners; however this dataset does not link prescribing data to clinical indication, which limits interpretation at a national level. Data from the NHS-Business Services Authority showed that between 1996 and 2000, total antibiotic prescribing in primary care declined year-on-year but that from 2000 onwards the trend was reversed [13]. This upsurge in prescribing occurred despite national guidance to avoid prescribing antibiotics for upper respiratory tract infections being issued by both the UK Department of Health’s Standing Medical Advisory Committee Sub-Group on Antimicrobial Resistance in 1998 and the National Institute for Health and Care Excellence in 2008 [14,15].

While the data on temporal trends in antibiotic prescribing are informative, a major limitation is that information on the clinical indication for which antibiotics are prescribed is not currently available, making it unfeasible to determine from this dataset whether treatment guidelines are being followed. However, assessment of the quality of antibiotic prescribing in primary care (based on whether the antibiotic prescribed for a given indication is consistent with available guidelines) can be undertaken using additional datasets such as the Clinical Practice Research Datalink and the Health Improvement Network which collect electronic medical records (including diagnoses and prescriptions issued) from general practices covering approximately 6%–7% of the population. Two recent studies using the Clinical Practice Research Datalink and the Health Improvement Network, respectively, have independently shown that for many practices the level of antibiotic prescribing for upper respiratory tract infections is above what is clinically justified based on national guidance, with the latter study showing that current levels are greater than those seen before recommendations were made to reduce it [17,18]. There was also substantial local variation in prescribing rates that is not explained by case-mix or variation in age or deprivation of patient populations. The reasons for overprescribing are not fully understood and are likely to be multi-factorial. Some primary care prescribers admit to being aware that their own prescribing may not always be clinically beneficial but are motivated to prescribe nonetheless through fear of what might happen should they withhold antibiotics, while some feel pressurised to prescribe antibiotics due to the high level of expectation of their patients. In recognition that attempts to reduce unnecessary antibiotic prescribing will involve inducing behaviour change on the part of both doctors and patients, the Public Health England and Department of Health Behavioural Insights Teams recently jointly undertook a literature review and analysis of prescribing behaviour. Their findings showed that most studies to date were not underpinned by psychological theory or behavioural science, and that further work using these approaches is required [19].

### 2.2. Antibiotic Prescribing in Secondary Care

The routine collection of data on prescribing in hospitals in England has been challenging due to the majority of hospitals not having systems in place for electronic recording of patient-level prescribing records. However, English hospital pharmacies provide aggregate monthly data on all medicines issued to in-patients, wards and clinics to IMS Health, a company providing information and technology services to the healthcare industry [20] (http://www.imshealth.com/portal/site/imshealth). IMS Health reimburses hospitals for these data and provides data handling and analytical support. IMS Health now has an agreement that enables it to share hospital antibiotic prescribing data with ESPAUR as well as with the former Antimicrobial Stewardship Sub-Group of the Advisory Committee on Antimicrobial Resistance and Healthcare-Associated Infections [6,21]. Trend analysis showed that 12-monthly total usage of antibiotics in English hospitals increased by 12.6% between September 2008 and 20012/2013, although there was marked variation in the temporal trends for use of different classes of antibiotics. For example, while use of first- and second-generation cephalosporins over the same period decreased by 25% and 41%, respectively, use of co-amoxiclav, carbapenems and piperacillin/tazobactam increased by 60%, 61% and 95%, respectively [21]. It was also noted that there was marked inter-hospital and geographical variation in prescribing [6,21].

## 3. Interventions to Improve Antibiotic Prescribing in England

The ESPAUR Report published in 2014 highlighted aspects of antibiotic prescribing in England that gave cause for concern, namely the increase in consumption of antibacterials between 2010 and 2013, the marked variations in prescribing between different regions and centres, and the increased use of critical broad-spectrum antibiotics in hospitals. To address these issues, the government’s advisory committee on Antimicrobial Resistance and Healthcare-Associated Infections recommended a reduction in antibacterial prescribing within both secondary and primary care, and published aspirational targets [22]. Additionally, the Health and Social Care Act 2008 has been updated to reflect the importance of optimizing antimicrobial use [7], with the Care Quality Commission who regulate provision of healthcare in England expecting all organizations providing healthcare to comply with the code of practice. This has driven the development and adoption of a variety of interventions to improve antimicrobial stewardship, with a particular focus on reducing the volume of antibiotic consumption and particularly the use of broad-spectrum antibiotics in both primary and secondary care.

## 4. Interventions in Primary Care

Medicines management pharmacists employed within Clinical Commissioning Groups and their Commissioning Support Units (CSUs) are responsible for providing prescribing advice to primary care prescribers, and this includes the development, implementation and audit of antimicrobial prescribing guidelines. National guidelines for the management of common infections in primary care that can be adopted for local use are published by Public Health England [23], but the wide variation in antibiotic prescribing data at both Clinical Commissioning Group and general practitioner level, suggest these guidelines are not consistently adopted by primary care prescribers. Hence, further interventions are required to improve and standardize antibiotic prescribing in primary care.

### 4.1. Quality Premium

To support Clinical Commissioning Group-led stewardship activity, detailed primary care antibiotic prescribing data are available from both the NHS-Business Services Authority Information Services Portal [24], and the PrescQIPP data Hub [25]. In 2015, NHS England published an antibiotic Quality Premium intended to reward Clinical Commissioning Groups for improvements in the quality of services they commission and for associated improvements in health outcomes and in reducing health inequalities [26]. Target values for a 1% reduction in the number of antibacterial prescription items and a 10% reduction in the proportion of broad-spectrum antibacterials (specifically co-amoxiclav, cephalosporins and quinolones) have been set for each Clinical Commissioning Group. To support the effective adoption and implementation of the antibiotic Quality Premium by commissioners, NHS England organized three national workshops in March 2015 that were attended by approximately 75% of Clinical Commissioning Groups [27]. The workshops were designed to raise awareness of antimicrobial resistance, promote stewardship activities and toolkits and share examples of successful stewardship activity within primary care.

### 4.2. The TARGET Toolkit

The TARGET (Treat Antibiotics Responsibly, Guidance, Education, Tools) toolkit is an on-line antimicrobial stewardship resource designed to help influence prescribers’ and patients’ personal attitudes, social norms and perceived barriers to optimal antibiotic prescribing [28,29]. Both a recent Patient Safety Alert issued jointly by NHS England and Public Health England [30] and antimicrobial stewardship guidelines issued by the National Institute for Health and Care Excellence [9] recommend the use of the TARGET resource to support effective stewardship. The TARGET toolkit includes a range of resources that can be used to support responsible antibiotic use by both prescribers and patients. Resources include a self-assessment checklist, training resources to help fulfil continuing professional development and revalidation requirements and a range of patient information leaflets including “Treating your infection”, “Get well soon without antibiotics” and “When should I worry?—Your guide to coughs, colds, earache and sore throats”. This resource is currently being adapted for use in community pharmacies to support self-care and for use by urgent care providers such as those working in walk-in centres.

## 5. Interventions in Secondary Care

### 5.1. Antimicrobial Stewardship Toolkit: Start Smart then Focus

**Figure 2:**
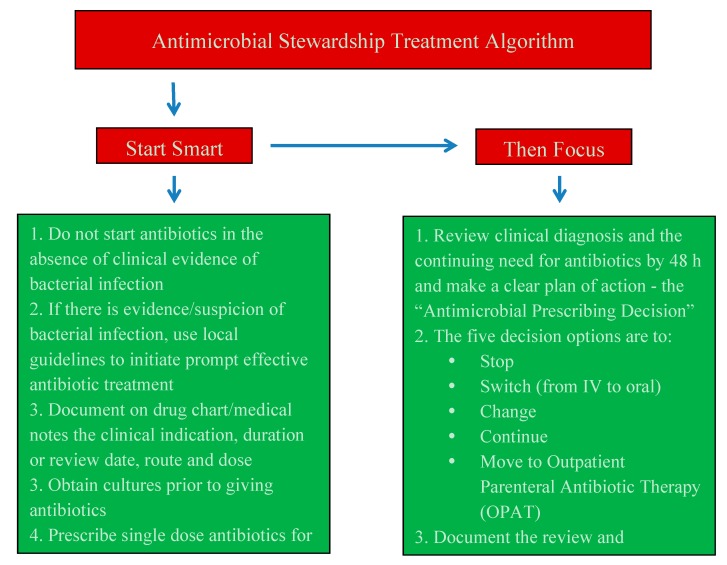
“Start Smart—Then Focus” antimicrobial stewardship guidance for hospitals.

Infection remains a significant cause of morbidity and mortality in critically ill patients. While the early recognition of infection and rapid initiation of broad-spectrum empirical antibiotic therapy is an essential component of the management of such patients, it is being increasingly emphasized that exposure of patients to antibiotics is a major driver for the emergence and spread of resistance and should be avoided where possible. Thus clinicians face the dilemma of trying to reconcile conflicting advice. To help address this issue, the Advisory Committee on Antimicrobial Resistance and Healthcare-Associated Infections has promulgated antimicrobial stewardship guidance for antibiotic prescribing in hospitals under the heading “Start Smart—Then Focus” [13,31]. This initiative has two inter-related aims, namely to improve the quality of the initial decision to prescribe an antibiotic combined with a second focus on the critical importance of formally reviewing antibiotic therapy at 48 h, based on the patient’s clinical response and diagnostic and antibiotic susceptibility test results from the laboratory. Put more succinctly, the core message of “Start Smart—Then Focus” is to give the right antibiotic at the right dose at the right time, followed by active review for all patients still on antibiotics 48–72 h after initiation of treatment [32]. The essentials components of this guidance are shown in Figure 2.

### 5.2. Validation of Antimicrobial Consumption Data

Data for antibiotic consumption in hospitals and primary care in England was published for the first time in 2014, as part of the ESPAUR Report [6]. The Report showed a total increase of 11.9% in prescribing to hospital in-patients over the four-year period (an average year-on-year increase of 3.5%, equivalent to 2.3 to 2.5 DDD per 1000 inhabitants per day). The Report also highlighted that there was considerable variability in both antibiotic resistance and antibiotic prescribing across England, with areas of high antimicrobial prescribing frequently associated with relatively high levels of resistance.

In England, IMS Health and Rx-info are commercial organizations that specialize in the provision of information, services and technology for the healthcare industry including the NHS. The hospital prescribing data used in the ESPAUR report was provided by IMS Health. Whilst both IMS Health and Rx-info have in-house quality assurance processes, the datasets have not however been externally validated. In June 2015, Public Health England validated the antimicrobial usage data of acute care Trusts in England as part of a quality improvement programme. To encourage participation, there is a requirement for hospitals to undertake data validation as a component of the national antimicrobial Quality Premium (financial reward) for Clinical Commissioning Groups [26].

### 5.3. Antimicrobial Self-Assessment Toolkit for Secondary Care

An evidence-based antimicrobial self-assessment toolkit (ASAT) for secondary care developed in collaboration with the Advisory Committee on Antimicrobial Resistance and Healthcare-Associated Infections and the Department of Health in 2012 has been updated and is now available as an on-line tool to enable individual secondary care organizations to benchmark themselves against the national average [33]. The main purpose of the ASAT is to allow individual acute organizations to self-evaluate and assess their antimicrobial stewardship practices. An ASAT evaluation allows hospitals to assess their performance against national guidelines and to identify areas for improvement so that they can address any gaps in service provision and delivery. The results from an evaluation could also be used to help hospitals to develop a strategy for addressing antimicrobial resistance and reducing healthcare-associated infections such as those caused by *C. difficile*.

## 6. Joint Primary and Secondary Care Antimicrobial Stewardship Activities

### 6.1. National Institute for Health and Care Excellence Guidance

The National Institute for Health and Care Excellence has recently published guidance on antimicrobial stewardship [9]. The target audience for this guidance was wide ranging encompassing hospital staff, general practitioners, dentists, podiatrists, pharmacists, community nurses, domiciliary care workers and care home staff, social workers and patients receiving antibiotics.

### 6.2. Antimicrobial Prescribing and Stewardship Competences

Education and training is an important aspect of improving stewardship. A multi-professional group led by the Advisory Committee on Antimicrobial Resistance and Healthcare-Associated Infections and Public Health England published national antimicrobial prescribing and stewardship competencies in 2014 [34]. The competences, which complement the generic competency framework for all prescribers [35] can be used by prescribers to develop their prescribing practice. It is also designed to provide clarity for regulators, education providers and professional bodies to inform standards, guidance and development of training.

## 7. Antimicrobial Stewardship for Patients and the Public

Public understanding of the issues around antimicrobial use and resistance is important for tackling antimicrobial resistance, as a patient’s expectations and demand can influence prescriber behaviour, particularly within primary and community care settings [19]. The antimicrobial resistance 2013 Special Eurobarometer Report involving 28 European Union (EU) member states revealed that knowledge of antibiotics among the UK population was generally higher than the EU average, with 52% of respondents in the UK aware that antibiotics are ineffective against viruses compared to the EU average of 40% [36]. However, colds and flu was still one of the five leading reasons for taking antibiotics reported by UK participants. Of further concern is the finding from a recent study undertaken by the Wellcome Trust that most people, if they had heard of antibiotic resistance at all, thought that it was their body that becomes resistant to antibiotics, rather than the bacteria that cause drug-resistant infections. This misconception makes people think that antibiotic resistance is someone else’s problem in the sense that if they feel they do not overuse or misuse antibiotics themselves, they mistakenly think resistance will not be a problem for them [37].

In light of the above, there is clearly an on-going need to increase awareness and knowledge among the public as to when antibiotics should or should not be taken. In 2015, Public Health England and the Department of Health published an analysis that identified key behaviours and drivers for antibiotic use in both the public and healthcare professionals [19,38]. Such information should help formulate interventions aimed at changing both public and professional attitudes towards antibiotic use. In addition, the National Institute for Health and Care Excellence is also currently in the process of developing public health guidance that will focus on changing people’s knowledge, attitudes and behaviours in relation to the use of antimicrobials, with publication of its guidance planned for early 2016.

### 7.1. European Antibiotic Awareness Day

The European Antibiotic Awareness Day organized by the European Centre for Disease Prevention and Control and held every year on 18 November is designed to encourage the responsible use of antibiotics [39]. In the UK range of educational materials including posters and leaflets to remind both the public and healthcare professionals of the need to reduce public expectation for antibiotics for coughs and colds is made available for local use. Whilst these campaigns have led to some increase in awareness that antibiotics are not effective for coughs and colds, effecting a prolonged change in public understanding of the proper use of antibiotics and its role in resistance is likely to continue to present a challenge for some time to come [40,41].

### 7.2. Antibiotic Guardian Campaign

The importance of public engagement rather than just awareness is increasingly being recognized. As a result, in 2014, Public Health England extended the European Antibiotic Awareness Day campaign objective in the UK from raising awareness to supporting people to take concrete personal and collective action to use antibiotics prudently. This took the form of the Antibiotic Guardian campaign, initiated as part of a drive to change behaviour by encouraging the public and professionals to make on-line pledges about how they would make better use of antibiotics. Drawing on evidence from behavioural science, implementation intentions (or if-then plans) were used to develop pledges in relation to antibiotic use. An exemplar pledge for a member of the public is “*The next time I have a cold or flu I will talk to the pharmacist first about how I can treat my symptoms rather than making a general practitioner appointment*”. At the time of writing, 13,772 individuals (including the three authors of this article) had signed up as Antibiotic Guardians. Pledges can be made on-line at http://antibioticguardian.com/.

## 8. Discussion

The critical role of antimicrobial stewardship in tackling the problem of antibiotic resistance is reflected in its inclusion as a key action in the UK five-year antibiotic resistance strategy. Antimicrobial stewardship is also an integral part of national strategies formulated by other countries including Australia [42] and the USA [43], as well as being included in the World Health Organization Global Action Plan [44,45]. With regard to the UK antibiotic resistance strategy, work on antimicrobial stewardship is on-going. In addition to the various activities outlined above, two surveys of primary and secondary care organizations have been undertaken to assess the implementation of the two national toolkits. These have been completed and the results, which highlighted poor implementation of the toolkits to date, will be published in the forthcoming ESPAUR Report in late 2015. It was the findings of these two surveys that resulted in the issue of a national patient safety alert for key leaders within primary and secondary care settings [27]. In the absence of new antibiotics active against antibiotic-resistant bacterial strains, antimicrobial stewardship will remain a critical tool in our efforts to contain resistance and preserve the clinical efficacy of currently available antibiotics.
